# *N*-(3-Chloro­phen­yl)succinimide

**DOI:** 10.1107/S1600536811026845

**Published:** 2011-07-09

**Authors:** B. S. Saraswathi, Sabine Foro, B. Thimme Gowda

**Affiliations:** a Department of Chemistry, Mangalore University, Mangalagangotri 574 199, Mangalore, India; b Institute of Materials Science, Darmstadt University of Technology, Petersenstrasse 23, D-64287 Darmstadt, Germany

## Abstract

In the title compound, C_10_H_8_ClNO_2_, the chloro­benzene and the essentially planar (r.m.s. deviation = 0.030 Å) pyrrolidine ring are tilted by 59.5 (1)° with respect to one another.

## Related literature

For our studies on the effects of substituents on the structures of *N*-(ar­yl)-amides, see: Bhat & Gowda (2000 ▶); Gowda *et al.* (1999 ▶, 2007 ▶); Saraswathi *et al.* (2010**a* ▶,b* ▶).
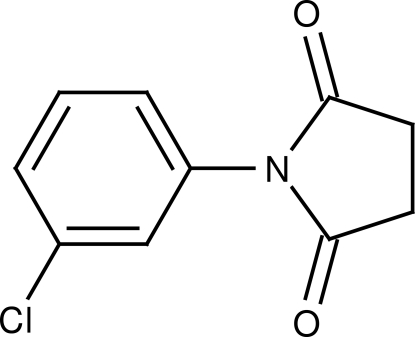


## Experimental

### 

#### Crystal data

C_10_H_8_ClNO_2_
*M* *_r_* = 209.62
Orthorhombic, 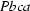

*a* = 12.884 (2) Å
*b* = 7.173 (1) Å
*c* = 20.805 (3) Å
*V* = 1922.7 (5) Å^3^
*Z* = 8
Mo *K*α radiation
μ = 0.37 mm^−1^
*T* = 293 K
0.46 × 0.12 × 0.09 mm

#### Data collection

Oxford Diffraction Xcalibur diffractometer with a Sapphire CCD detector
Absorption correction: multi-scan (*CrysAlis RED*; Oxford Diffraction, 2009 ▶) *T*_min_ = 0.849, *T*_max_ = 0.968
6087 measured reflections
1755 independent reflections
1163 reflections with *I* > 2σ(*I*)
*R*_int_ = 0.044

#### Refinement

*R*[*F* ^2^ > 2σ(*F* ^2^)] = 0.082
*wR*(*F* ^2^) = 0.137
*S* = 1.33
1755 reflections
127 parameters
H-atom parameters constrained
Δρ_max_ = 0.32 e Å^−3^
Δρ_min_ = −0.46 e Å^−3^

### 

Data collection: *CrysAlis CCD* (Oxford Diffraction, 2009 ▶); cell refinement: *CrysAlis RED* (Oxford Diffraction, 2009 ▶); data reduction: *CrysAlis RED*; program(s) used to solve structure: *SHELXS97* (Sheldrick, 2008 ▶); program(s) used to refine structure: *SHELXL97* (Sheldrick, 2008 ▶); molecular graphics: *PLATON* (Spek, 2009 ▶); software used to prepare material for publication: *SHELXL97*.

## Supplementary Material

Crystal structure: contains datablock(s) I, global. DOI: 10.1107/S1600536811026845/bt5570sup1.cif

Structure factors: contains datablock(s) I. DOI: 10.1107/S1600536811026845/bt5570Isup2.hkl

Supplementary material file. DOI: 10.1107/S1600536811026845/bt5570Isup3.cml

Additional supplementary materials:  crystallographic information; 3D view; checkCIF report

## Figures and Tables

**Fig. 1.**
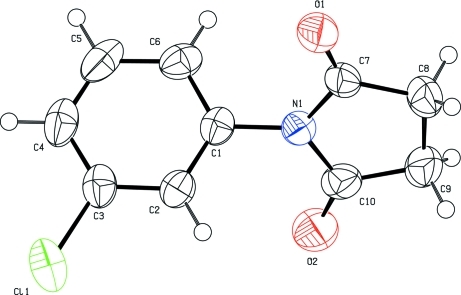
Molecular structure of (I), showing the atom labeling. Displacement ellipsoids drawn at the 50% probability level.

**Fig. 2.**
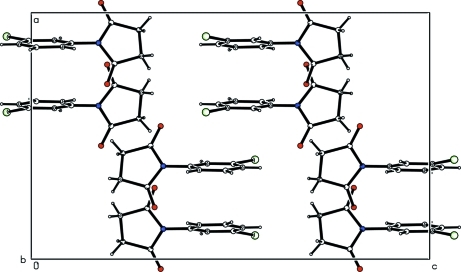
Molecular packing of (I).

## supplementary crystallographic information

### Comment

As a part of our studies on the effects of ring and side chain substitutions on the structures and other aspects of biologically significant compounds (Bhat & Gowda, 2000; Gowda *et al.*, 1999, 2007; Saraswathi *et al.*, 2010**a*,b*), the crystal structure of *N*-(3-chlorophenyl)succinimide has been determined (Fig. 1). In the structure, the molecule is non-planar with the benzene and pyrrolidine rings tilted by 59.5 (1)° with respect to one another, compared to the values of 69.5 (1)° in *N*-(2-chlorophenyl)- succinimide (Saraswathi *et al.*, 2010*a*) and 52.5 (1)° in *N*-(3-methylphenyl)succinimide (Saraswathi *et al.*, 2010*b*).

The torsional angles of the groups, C2 - C1 - N1 - C7, C6 - C1 - N1 - C7, C2 - C1 - N1 - C10 and C6 - C1 - N1 - C10 in the molecule are -117.5 (5), 61.9 (5), 57.7 (5)° and -123.0 (4), respectively, while the torsional angles of the groups, O1 - C7 - N1 - C1, C8 - C7 - N1 - C1, O2 - C10 - N1 - C1 and C9 - C10 - N1 - C1 are 0.5 (6), -178.4 (4), 2.7 (7) and -177.6 (4)°, respectively.

The packing of molecules into layered chains along *a*-axis is shown in Fig. 2.

### Experimental

The solution of succinic anhydride (0.02 mole) in toluene (25 ml) was treated dropwise with the solution of 3-chloroaniline (0.02 mole) also in toluene (20 ml) with constant stirring. The resulting mixture was stirred for one hour and set aside for an additional hour at room temperature for the completion of reaction. The mixture was then treated with dilute hydrochloric acid to remove the unreacted 3-chloroaniline. The resultant solid *N*-(3-chlorophenyl)succinamic acid was filtered under suction and washed thoroughly with water to remove the unreacted succinic anhydride and succinic acid. It was recrystallized to constant melting point from ethanol.

*N*-(3-chlorophenyl)succinamic acid was heated for 2 h and then allowed to cool slowly to room temperature to get the compound, *N*-(3-chlorophenyl)succinimide. The purity of the compound was checked and characterized by its infrared spectra.

Needle like colourless single crystals of the compound used in X-ray diffraction studies were grown in ethanolic solution by a slow evaporation at room temperature.

### Refinement

The H atoms were positioned with idealized geometry using a riding model with the aromatic C—H = 0.93 Å and methylene C—H = 0.97 Å and were refined with isotropic displacement parameters  set to 1.2 times of the *U*_eq_ of the parent atom.

### Crystal data

**Table:**

C_10_H_8_ClNO_2_	*F*(000) = 864
*M**_r_* = 209.62	*D*_x_ = 1.448 Mg m^−^^3^
Orthorhombic, *P**b**c**a*	Mo *K*α radiation, λ = 0.71073 Å
Hall symbol: -P 2ac 2ab	Cell parameters from 1436 reflections
*a* = 12.884 (2) Å	θ = 2.8–27.8°
*b* = 7.173 (1) Å	µ = 0.37 mm^−^^1^
*c* = 20.805 (3) Å	*T* = 293 K
*V* = 1922.7 (5) Å^3^	Needle, colourless
*Z* = 8	0.46 × 0.12 × 0.09 mm

### Data collection

**Table:**

Oxford Diffraction Xcalibur diffractometer with a Sapphire CCD detector	1755 independent reflections
Radiation source: fine-focus sealed tube	1163 reflections with *I* > 2σ(*I*)
graphite	*R*_int_ = 0.044
Rotation method data acquisition using ω scans	θ_max_ = 25.4°, θ_min_ = 3.2°
Absorption correction: multi-scan (*CrysAlis RED*; Oxford Diffraction, 2009)	*h* = −9→15
*T*_min_ = 0.849, *T*_max_ = 0.968	*k* = −6→8
6087 measured reflections	*l* = −25→22

### Refinement

**Table:**

Refinement on *F*^2^	Primary atom site location: structure-invariant direct methods
Least-squares matrix: full	Secondary atom site location: difference Fourier map
*R*[*F*^2^ > 2σ(*F*^2^)] = 0.082	Hydrogen site location: inferred from neighbouring sites
*wR*(*F*^2^) = 0.137	H-atom parameters constrained
*S* = 1.33	*w* = 1/[σ^2^(*F*_o_^2^) + (0.0157*P*)^2^ + 3.1516*P*] where *P* = (*F*_o_^2^ + 2*F*_c_^2^)/3
1755 reflections	(Δ/σ)_max_ = 0.001
127 parameters	Δρ_max_ = 0.32 e Å^−^^3^
0 restraints	Δρ_min_ = −0.46 e Å^−^^3^

### Special details

**Table:**

Geometry. All e.s.d.'s (except the e.s.d. in the dihedral angle between two l.s. planes) are estimated using the full covariance matrix. The cell e.s.d.'s are taken into account individually in the estimation of e.s.d.'s in distances, angles and torsion angles; correlations between e.s.d.'s in cell parameters are only used when they are defined by crystal symmetry. An approximate (isotropic) treatment of cell e.s.d.'s is used for estimating e.s.d.'s involving l.s. planes.
Refinement. Refinement of *F*^2^ against ALL reflections. The weighted *R*-factor *wR* and goodness of fit *S* are based on *F*^2^, conventional *R*-factors *R* are based on *F*, with *F* set to zero for negative *F*^2^. The threshold expression of *F*^2^ > σ(*F*^2^) is used only for calculating *R*-factors(gt) *etc*. and is not relevant to the choice of reflections for refinement. *R*-factors based on *F*^2^ are statistically about twice as large as those based on *F*, and *R*- factors based on ALL data will be even larger.

### Fractional atomic coordinates and isotropic or equivalent isotropic displacement parameters (Å^2^)

**Table:**

	*x*	*y*	*z*	*U*_iso_*/*U*_eq_	
C1	0.1240 (3)	0.3985 (6)	0.39944 (19)	0.0406 (10)	
C2	0.1122 (3)	0.2512 (6)	0.44197 (19)	0.0427 (10)	
H2	0.1035	0.1300	0.4269	0.051*	
C3	0.1135 (3)	0.2881 (7)	0.5070 (2)	0.0474 (12)	
C4	0.1279 (3)	0.4651 (8)	0.5302 (2)	0.0572 (13)	
H4	0.1292	0.4874	0.5743	0.069*	
C5	0.1403 (3)	0.6090 (7)	0.4874 (2)	0.0590 (14)	
H5	0.1504	0.7294	0.5028	0.071*	
C6	0.1381 (3)	0.5785 (6)	0.4213 (2)	0.0489 (12)	
H6	0.1459	0.6769	0.3926	0.059*	
C7	0.2069 (4)	0.3968 (6)	0.2912 (2)	0.0437 (11)	
C8	0.1764 (4)	0.3477 (7)	0.2235 (2)	0.0557 (13)	
H8A	0.2260	0.2619	0.2047	0.067*	
H8B	0.1725	0.4586	0.1970	0.067*	
C9	0.0704 (4)	0.2568 (7)	0.2297 (2)	0.0600 (14)	
H9A	0.0203	0.3189	0.2023	0.072*	
H9B	0.0738	0.1263	0.2177	0.072*	
C10	0.0405 (4)	0.2769 (6)	0.2994 (2)	0.0498 (12)	
N1	0.1227 (3)	0.3623 (4)	0.33150 (16)	0.0408 (9)	
O1	0.2895 (2)	0.4592 (4)	0.30949 (15)	0.0591 (9)	
O2	−0.0397 (3)	0.2302 (5)	0.32476 (16)	0.0706 (10)	
Cl1	0.09625 (11)	0.1056 (2)	0.56153 (6)	0.0722 (5)	

### Atomic displacement parameters (Å^2^)

**Table:**

	*U*^11^	*U*^22^	*U*^33^	*U*^12^	*U*^13^	*U*^23^
C1	0.033 (2)	0.048 (3)	0.041 (2)	0.002 (2)	−0.0020 (19)	−0.006 (2)
C2	0.042 (3)	0.042 (2)	0.043 (3)	0.004 (2)	−0.001 (2)	−0.006 (2)
C3	0.036 (3)	0.067 (3)	0.039 (3)	0.002 (2)	−0.001 (2)	−0.005 (2)
C4	0.042 (3)	0.085 (4)	0.044 (3)	−0.004 (3)	0.003 (2)	−0.021 (3)
C5	0.041 (3)	0.065 (3)	0.071 (3)	−0.008 (3)	0.006 (3)	−0.035 (3)
C6	0.038 (3)	0.045 (3)	0.064 (3)	−0.004 (2)	0.005 (2)	−0.008 (2)
C7	0.055 (3)	0.035 (2)	0.042 (2)	0.003 (2)	0.000 (2)	0.005 (2)
C8	0.076 (3)	0.052 (3)	0.040 (3)	0.002 (3)	−0.003 (2)	0.007 (2)
C9	0.081 (4)	0.053 (3)	0.046 (3)	−0.006 (3)	−0.018 (3)	0.003 (2)
C10	0.057 (3)	0.039 (3)	0.053 (3)	−0.003 (2)	−0.013 (3)	0.006 (2)
N1	0.044 (2)	0.040 (2)	0.0386 (19)	−0.0028 (17)	−0.0024 (17)	0.0015 (17)
O1	0.053 (2)	0.069 (2)	0.055 (2)	−0.0133 (18)	0.0055 (17)	0.0002 (17)
O2	0.057 (2)	0.087 (3)	0.068 (2)	−0.024 (2)	−0.011 (2)	0.003 (2)
Cl1	0.0824 (9)	0.0924 (10)	0.0418 (6)	0.0078 (8)	0.0035 (7)	0.0082 (7)

### Geometric parameters (Å, °)

**Table:**

C1—C6	1.380 (6)	C7—O1	1.214 (5)
C1—C2	1.387 (6)	C7—N1	1.393 (5)
C1—N1	1.437 (5)	C7—C8	1.505 (6)
C2—C3	1.379 (6)	C8—C9	1.518 (7)
C2—H2	0.9300	C8—H8A	0.9700
C3—C4	1.371 (6)	C8—H8B	0.9700
C3—Cl1	1.746 (5)	C9—C10	1.507 (6)
C4—C5	1.374 (7)	C9—H9A	0.9700
C4—H4	0.9300	C9—H9B	0.9700
C5—C6	1.393 (6)	C10—O2	1.208 (5)
C5—H5	0.9300	C10—N1	1.395 (5)
C6—H6	0.9300		
			
C6—C1—C2	121.2 (4)	N1—C7—C8	108.5 (4)
C6—C1—N1	119.6 (4)	C7—C8—C9	104.9 (4)
C2—C1—N1	119.2 (4)	C7—C8—H8A	110.8
C3—C2—C1	118.6 (4)	C9—C8—H8A	110.8
C3—C2—H2	120.7	C7—C8—H8B	110.8
C1—C2—H2	120.7	C9—C8—H8B	110.8
C4—C3—C2	121.7 (4)	H8A—C8—H8B	108.8
C4—C3—Cl1	118.9 (4)	C10—C9—C8	105.7 (4)
C2—C3—Cl1	119.4 (4)	C10—C9—H9A	110.6
C3—C4—C5	118.9 (4)	C8—C9—H9A	110.6
C3—C4—H4	120.6	C10—C9—H9B	110.6
C5—C4—H4	120.6	C8—C9—H9B	110.6
C4—C5—C6	121.4 (5)	H9A—C9—H9B	108.7
C4—C5—H5	119.3	O2—C10—N1	124.2 (4)
C6—C5—H5	119.3	O2—C10—C9	127.8 (4)
C1—C6—C5	118.3 (4)	N1—C10—C9	108.0 (4)
C1—C6—H6	120.8	C7—N1—C10	112.4 (4)
C5—C6—H6	120.8	C7—N1—C1	123.3 (3)
O1—C7—N1	124.1 (4)	C10—N1—C1	124.1 (4)
O1—C7—C8	127.5 (4)		
			
C6—C1—C2—C3	0.8 (6)	C8—C9—C10—N1	−2.7 (5)
N1—C1—C2—C3	−179.9 (4)	O1—C7—N1—C10	−175.1 (4)
C1—C2—C3—C4	−1.1 (7)	C8—C7—N1—C10	6.0 (5)
C1—C2—C3—Cl1	178.8 (3)	O1—C7—N1—C1	0.5 (6)
C2—C3—C4—C5	0.6 (7)	C8—C7—N1—C1	−178.4 (4)
Cl1—C3—C4—C5	−179.3 (3)	O2—C10—N1—C7	178.3 (4)
C3—C4—C5—C6	0.3 (7)	C9—C10—N1—C7	−2.0 (5)
C2—C1—C6—C5	0.1 (6)	O2—C10—N1—C1	2.7 (7)
N1—C1—C6—C5	−179.2 (4)	C9—C10—N1—C1	−177.6 (4)
C4—C5—C6—C1	−0.6 (7)	C6—C1—N1—C7	61.9 (5)
O1—C7—C8—C9	173.9 (4)	C2—C1—N1—C7	−117.5 (4)
N1—C7—C8—C9	−7.3 (5)	C6—C1—N1—C10	−123.0 (4)
C7—C8—C9—C10	5.9 (5)	C2—C1—N1—C10	57.7 (5)
C8—C9—C10—O2	176.9 (5)		
